# Metalloporphyrin Dimers Bridged by a Peptoid Helix: Host-Guest Interaction and Chiral Recognition

**DOI:** 10.3390/molecules23112741

**Published:** 2018-10-24

**Authors:** Yen Jea Lee, Boyeong Kang, Jiwon Seo

**Affiliations:** Department of Chemistry, School of Physics and Chemistry, Gwangju Institute of Science and Technology, Gwangju 61005, Korea; leeyeonjae@gist.ac.kr (Y.J.L.); boyeongkang2022@u.northwestern.edu (B.K.)

**Keywords:** porphyrin, peptoid, porphyrin tweezers, co-facial porphyrin, host-guest, chiral recognition, exciton-coupled circular dichroism

## Abstract

Co-facial porphyrins have been designed to construct porphyrin tweezers with versatile molecular recognition capabilities. In this study, we synthesized metalloporphyrin–peptoid conjugates (MPPCs) displaying two metalloporphyrins on a peptoid scaffold with either achiral unfolded (**1**) or helical (**2** and **3**) secondary structures. Host–guest complexation of MPPCs was realized with various guests of different lengths and basicities, and the extent of complexation was measured by UV-vis and circular dichroism (CD) spectroscopic titration. Intermolecular and intramolecular chirality induction were observed on achiral and chiral peptoid backbones, respectively. Spectroscopic data indicated that a broad scope of achiral guests can be recognized by chiral **2**; in particular, longer and more flexible guests were seen to bind more tightly on **2**. In addition, chiral **2** provided a distinct CD couplet with dl-, d-, or l-Lys-OMe, which was a result of the diastereomeric host–guest complex formation. Our results indicated that MPPCs can recognize, contrast, and analyze various achiral, chiral, or racemic molecules. Based on co-facial metalloporphyrins present on peptoid scaffolds, we developed a novel class of porphyrin tweezers, which can be further utilized in asymmetric catalysis, molecular sensing, and drug delivery.

## 1. Introduction

In photosynthetic light-harvesting complexes, the distance between numerous pigments and their arrangement are precisely controlled by a helical protein matrix. This spatial regulation is directly related to the highly efficient natural photosynthetic process [1,2]. Inspired by Nature, multiporphyrin arrays have been constructed as artificial light-harvesting systems [3,4,5,6] or as molecular photonic or electronic wires [7,8,9]. Various efforts have been made to improve the function of these multiporphyrin systems, including the construction of co-facial porphyrins on molecular scaffolds such as neucleotides [10], anthrancenics [10,11], peptide [12,13], and other peptidomimetics [14,15,16]. 

Co-facial porphyrins have been actively used in the design of porphyrin tweezers. Because of their outstanding photophysical properties such as strong visible-light absorption in the Soret band (~410 nm) and intense fluorescence emission (500–700 nm), porphyrin tweezers can readily detect environmental changes at low concentrations (below μM levels) [17]. Porphyrin tweezers can be engineered to recognize and differentiate chirality while maintaining this intrinsic photophysical sensitivity, and therefore, are employed frequently as a chiral sensing reagent [17,18,19,20,21,22]. By the complexation of an achiral porphyrin tweezer with a chiral guest, the chirality of the guest can be transferred to the host; subsequently, the interporphyrin arrangement prefers a certain chiral twist depending on the asymmetric configuration of the guest. Furthermore, the chiral interporphyrin twist results in an exciton-coupled circular dichroism (ECCD) signal; this phenomenon is called intermolecular chirality induction [17,23]. 

Chiral co-facial porphyrins are interesting molecular tweezers by virtue of their enantioselective binding with chiral guests. By connecting two porphyrins with chiral linkers, such as 1,1′-bi-2-naphthol (BINOL) [23], chiral diamine [24], or benzyl ester [25], a pre-organized chiral interporphyrin twist can be maintained through intramolecular chirality induction. The chiral porphyrin tweezer recognizes and differentiates chiral guests such as amino acids [26] or diamines [27], and this leads to enantioselective host–guest binding.

Along with chirality, conformational flexibility of a porphyrin tweezer is essential for its complexation with various substrates [11,18,25,28]. If the porphyrin tweezer is extremely rigid, it cannot form an “induced fit” to accommodate a wide variety of guests [29]. In contrast, if a porphyrin tweezer is extremely flexible, it will result in poor binding affinity or nonspecific binding [30]. Because few scaffolds are capable of controlling the backbone conformational flexibility, studies concerning the effect of backbone flexibility on the host–guest complexation are scarce.

Previously, we reported porphyrin–peptoid conjugates (PPCs) capable of modulating the interporphyrin arrangement and distance by the position-specific incorporation of porphyrins on a helical peptoid [14,15,16]. Peptoids are sequence specific peptidomimetics based on the *N*-substituted glycine backbone. By inserting a monomer sequentially, peptoids can form secondary structures such as polyproline type I (PPI)-like helix [31], ribbons [32], and square helix [33]. In related studies, PPI-like peptoid helix possessing α-chiral aromatic amine side chains exhibits a pitch length of ~6 Å, and a periodicity of three residues per turn [31]. More recently, the modulation of peptoid helicity using the sergeant-and-solider effect was demonstrated [34]. The established solid phase peptoid synthesis method [35] and efficient post-synthetic modifications [36,37,38,39,40,41] afforded peptoids with greater structural diversity, thereby leading to their application in various functional entities including bioactive agents [42,43,44,45,46,47], biomimetic materials [48,49,50,51], sensors [52,53], and metal-chelating ligands [48,54,55,56,57,58,59].

**Scheme 1:**
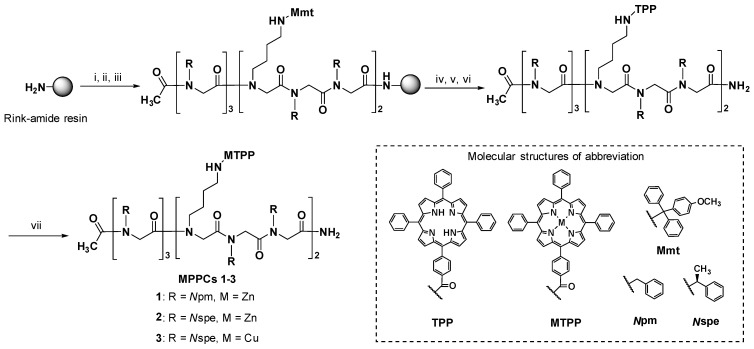
Synthesis of metalloporphyrin–peptoid conjugates (MPPCs) **1−3**. *Reagents and Conditions*: (i) bromoacetic acid (1.2 M), DIC, DMF, microwave irradiation, 35 °C, 1.5 min; (ii) primary amines (1.5–1.0 M), NMP, microwave irradiation, 80 °C, 4 min; (i) and (ii) were repeated until a desired peptoid sequence was obtained; (iii) acetic anhydride (8.4 M), pyridine, DMF, room temperature (rt), 2 h; (iv) TFA:TIS:CH_2_Cl_2_ = 0.75:5:94.25 (*v*/*v*/*v*), rt, 3 min, repeated over 12 times; (v) TPP-NHS, *i*-Pr_2_NEt, CH_2_Cl_2_, rt, overnight; (vi) 95% TFA in CH_2_Cl_2_, rt; (vii) Zn(OAc)_2_, *i*-Pr_2_NEt (or Cu(OAc)_2_ without base), MeOH/CH_2_Cl_2_, rt, overnight.

**Figure 1:**
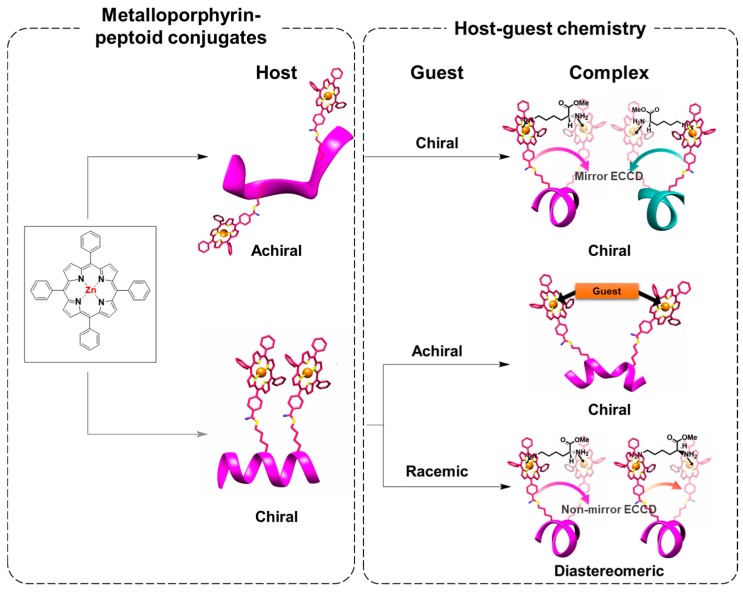
Schematic illustration of host–guest complexation between metalloporphyrin-peptoid conjugate (MPPC) and guest. Specific interactions between achiral host-chiral guest, chiral host-achiral guest, and chiral host-chiral guest are investigated in this study.

Co-facial porphyrins on porphyrin-peptoid conjugates can act as molecular tweezers whose structural characteristics (e.g., chirality, spatial arrangement, and conformational flexibility) can be influenced by peptoid scaffolds [16]. This type of structural controllability can be rarely found in other porphyrin tweezers. In addition, the tertiary amide backbone of peptoids and their dynamic cis-trans isomerism renders the porphyrin tweezers moderately flexible to adopt various guests. In this study, we synthesized three new MPPCs with different backbone chirality and metals (Zn or Cu) (Scheme 1). Using UV-vis and circular dichroism (CD) spectroscopy, three types of interactions between the MPPC host and guest molecules were investigated: (1) achiral host–chiral guest, (2) chiral host–achiral guest, and (3) chiral host–chiral guest complexations (Figure 1). The structural and chemical features (i.e., length and basicity) of various bidentate guests were evaluated upon their complexation with MPPCs. Moreover, using chiral MPPCs, chiral differentiation and racemate analysis were performed. Our MPPCs provided an insight into the design of a moderately flexible chiral porphyrin tweezer that can recognize a broad scope of guest molecules efficiently.

## 2. Results and Discussion

### 2.1. Synthesis of MPPCs

Three MPPCs of different peptoid backbones (achiral peptoid backbone with benzylamine, *N*pm and right-handed helix with (*S*)-(−)-1-phenylethylamine, *N*spe) [31,60] and different metal ions were synthesized (Scheme 1). The peptoid sequence was synthesized following the standard solid-phase submonomer synthesis protocol [35], and tetraphenylporphyrins (TPPs) were conjugated on the resin-bound peptoid employing the method (Scheme 1) reported previously [14,15]. Metal incorporation on the porphyrin ring was performed with zinc acetate or copper acetate in a methanol/dichloromethane mixture. The excess metal salt was removed using an SPE cartridge (see experimental for the detailed procedures). After the removal of excess metal salts, each MPPC of purity exceeding 98% was analyzed by LC-MS based on the chromatogram integration by UV detection at 220 nm (Figure S1).

### 2.2. Achiral Host-Chiral Guest Complexation: Intermolecular Chirality Induction

**Figure 2:**
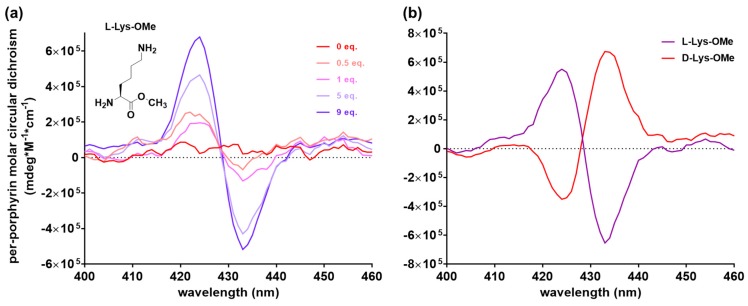
Circular dichroism (CD) spectra of **1** (1 μM) in CH_2_Cl_2_ at 20 °C upon the addition of l- or d-Lys-OMe. (**a**) l-Lys-OMe was added from 0 to 9 equivalents. (**b**) CD spectra of **1** with l- or d-Lys-OMe (17 equivalent each) were compared.

Two pigments arranged in a chiral environment exhibit ECCD [61]. Owing to the achiral peptoid scaffold, two porphyrins of **1** cannot generate an ECCD signal [14,60]. If a chiral guest is caught between the two porphyrins of **1**, the intercalation of the chiral guest can induce the chiral orientation between two porphyrins, thereby generating an ECCD signal [17,18]. This type of chirality transfer from a guest to a host is called chirality induction. l- or d-Lys-OMe were used to verify that **1** can pick up one guest as a molecular tweezer, and was analyzed by CD spectroscopy (Figure 2). With the addition of l-Lys-OMe, the ECCD signal of **1** appeared, with the maximum at 425 nm and minimum at 432 nm, and it was a negative Cotton effect indicating a left-handed interaction between porphyrins (Figure 2a). In the case of d-Lys-OMe, the ECCD signal exhibited a mirror image that indicates d-Lys-OMe inducing a right-handed orientation of porphyrins (Figure 2b).

### 2.3. Chiral Host–Achiral Guest Complexation

The peptoid helix is a useful tool to control the interaction between porphyrins [14,15,16]. We employed a right-handed helix by incorporating *N*spe into the peptoid sequence to develop chiral porphyrin tweezers, **2** and **3** [14,15,16]. The CD spectra of **2** were scanned from 190–260 nm (Figure S2), and it indicated that **2** maintained PPI-like helix in ACN, MeOH, and ACN/MeOH mixtures.

The molecular conformation of the peptoid backbone is restricted at low temperatures. Subsequently, the co-facial porphyrins on peptoid exhibited strong interaction. Therefore, the temperature dependence of the ECCD signal from the porphyrins indicates whether the porphyrins are facing each other on a molecular scaffold. The temperature was controlled from 20 °C to −10 °C when CD spectra of **2** were obtained from 400 nm to 450 nm (Figure S2). With the low temperature (−10 °C), the ECCD signal of **2** became stronger. This suggests that the facial arrangement of zinc porphyrins was introduced well on the peptoid helix of **2**.

As shown in Figure S2, **2** demonstrated a positive Cotton effect without any guest molecules owing to the intramolecular chirality induction afforded by a right-handed helical peptoid (Figure S2a). Compared to the porphyrin tweezers bearing alkyl chain or linkers reported previously, the peptoid helix can provide a spatially defined environment between two porphyrins. Therefore, the co-facial porphyrins of **2** was expected to exhibit different binding preferences depending on the characteristics of the guest, such as length and basicity.

**Figure 3:**
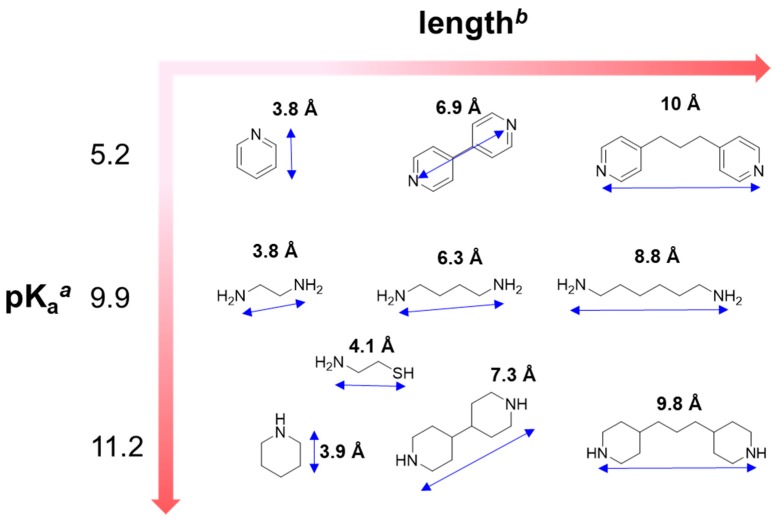
Guest molecules containing nitrogen atom(s). ^a^ The pK_a_ values of pyridine [62], ethylenediamine [63], and piperidine [64] are shown on the left side of Figure. ^b^ The lengths of the molecules were computationally measured from nitrogen atom to nitrogen (or sulfur) atom for bidentate ligands and from nitrogen to hydrogen atom for pyridine and piperidine after energy minimization using the MM2 force field in Chem3D 16.0.

The host–guest complexations of **2** with guest molecules of different lengths and basicities (pK_a_) were evaluated using CD and UV-vis spectroscopy. Various guests containing pyridyl, amino, piperidinyl, and thiol (Figure 3) groups were used to confirm the characteristics of guests that could be recognized by **2**. During the spectroscopic titration of **2**, the characteristic ECCD signal decreased owing to the achiral guest intercalation weakening the chiral interaction of porphyrins.

The modulation of intramolecular chirality induction by achiral bidentate guests revealed a spectroscopic shift depending on the length of the guests. Without any guest, **2** exhibited its maximal absorption at 427 nm. With ethylenediamine or 1,4-diaminobutane, the maxima of the UV-vis spectra of **2** were blue shifted (Figure S3d–e, Table 1). These short guests may place porphyrins close together, inducing face-to-face-type porphyrin arrangement (i.e., *H*-aggregation). Longer guests such as 1, 6-diaminohexane did not exhibit a similar spectral shift. In the case of 4,4′-trimethylenedipiperidine, which is longer and bulkier than 1,6-diaminobutane, a bathochromic shift was observed with **2** at 429 nm (Table 1). Bulkier and longer guests are also recognized by **2**, and an edge-to-edge-type porphyrin arrangement (i.e., *J*-aggregation) was formed. Therefore, **2** can distinguish the length and bulkiness of the guests effectively by providing spectral shifts. 

Clear isosbestic points on spectroscopic titration indicate a formation of a well-defined supramolecular complex between a host and guest [23,65,66]. Except for pyridine, most guests tested indicated clear isosbestic points in spectroscopic titration. When 4,4-dipyridyl and 4,4′-trimethylenedipyridine were used as guests, a clear isosbestic point near 423 nm (Figure S3b,c) appeared, indicating that a 1:1 binding is predominant. Otherwise, diamine guests exhibited different numbers of isosbestic points; nevertheless, this type of guests demonstrated well-defined isosbestic points. For example, 1,4-diaminobutane and 1,6-diaminohexane revealed three and two isosbestic points, respectively (Figure S3e,f); therefore, the 1:1 to 1:3 binding mode for 1,4-diaminobutane, and 1:1 and 1:2 binding modes for 1,6-diaminohexane can be expected. Other piperidinyl guests exhibit one isosbestic point in each titration data near 425 nm, thus indicating the dominance of 1:1 complexation (Figure S3h,i). Interestingly, the first isosbestic points near 423–427 nm appeared with most guests on the spectroscopic titration; therefore, we can assume that 1:1 binding complexations with the tested guests possess a similar structural pattern. Therefore, the decreasing absorbance at 416 nm of spectroscopic titration can be employed universally to calculate the dissociation constants (binding constants) for each complexation.

**Figure 4:**
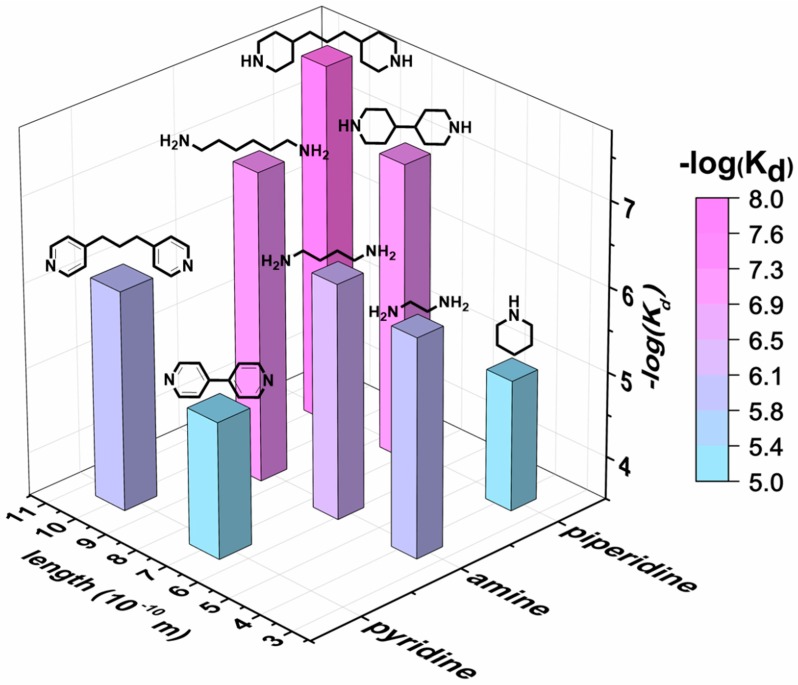
Comparison of K_d_ values for the complexation of **2** and guests.

The dissociation constant, K_d_ was calculated with the UV-vis titration with a broad scope of dinitrogen guests. As the addition of guests increased, the absorbance at 416 nm (a shoulder of Soret band) decreased (Figure S3). The decreasing absorbance at 416 nm was fitted using nonlinear regression (detail in experimental section). Consequently, the longer, more flexible and basic are the guests, the stronger is the binding (Figure 4). According to a structural study of porphyrin-peptoid conjugates by Kang and co-workers, the interporphyrin distance was expected to be ~6 Å based on molecular simulation and spectroscopic analysis [15]. Therefore, we expected that the optimal length of guest will be approximately 6 Å, e.g., 4,4-dipyridyl and 1,4-diaminobutane. However, longer guests such as 4,4′-trimethylenedipyridyl, 1,6-diaminohexane, and 4,4′-trimethylene-dipiperidine demonstrated stronger binding with lower K_d_ compared to shorter guests such as 4,4′-dipyridyl, 1,4-diaminobutane, and 4,4′-bipiperidine (Figure 4 and Table 1). Considering that two porphyrins were connected onto the peptoid backbone through a relatively flexible *n*-butyl linkage, we speculated that, because of its inherent flexibility [15,16], **2** binds effectively by adopting longer and more flexible guests that possess higher degrees of freedom. Thermodynamically, the zinc ion creates a strong coordination with basic nitrogen possessing higher pK_a_. This explains why piperidinyl guests display lower K_d_ values than pyridyl guests.

**Figure 5:**
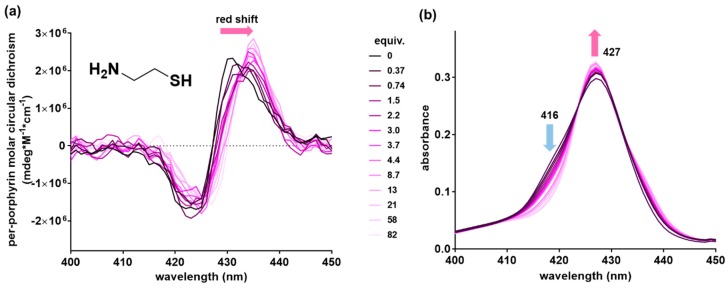
Titration of **2** (0.27 μM) with cysteamine in CH_2_Cl_2_ at 20 °C monitored by (**a**) CD and (**b**) UV-vis spectroscopy.

Cysteamine is a drug for cystinosis that results from overaccumulation of cysteine (or disulfide-linked dicysteine) in the cells, and is a potent candidate for treating neurodegenerative diseases such as Huntington’s and Parkinson’s diseases [67]. Therefore, we chose cysteamine as a biologically interesting guest for **2**. The complexation of **2** and cysteamine was evaluated by CD and UV-vis spectroscopy (Figure 5).

Cysteamine with one amino group exhibited a K_d_ of 9.5 × 10^−7^ M, which was similar to the value of ethylenediamine. We conclude that the thiol of cysteamine also binds to the zinc porphyrin of **2**. CD and UV-vis spectra with cysteamine demonstrated distinguishable CD profiles (Figure 5), compared to other spectra with nitrogen containing guests (Figure S3). As the equivalents of cysteamine increased, the CD maxima were shifted from 430 nm to 435 nm, and the spectrum intensity was increased. This difference can be considered as a sulfur binding effect on the porphyrin tweezer. We expect MPPCs to be applied as a sensor platform for thiol containing molecules.

The metal species in a metalloporphyrin tweezer is important for the selection of interaction between the metal and guests. Copper (II) was known to strongly bind with oxygen [68]; however, copper porphyrin-peptoid conjugate **3** did not show specific binding with 1,4-butanediol nor with 4,4′-dipyridyl (Figure S5). The binding event of **3** with 1,4-butanediol could not be clearly demonstrated when UV or CD spectroscopy were used (Figure S5b). Therefore, we focused on the host–guest effect between the zinc porphyrin peptoid conjugate **2** and nitrogen-containing guests in this study.

### 2.4. Chiral Host–Chiral Guest Complexation

**Figure 6:**
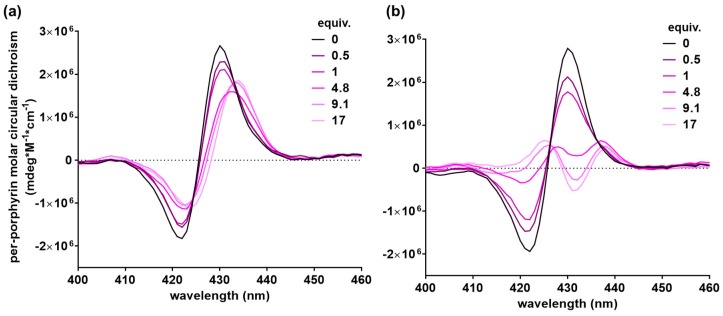
CD and UV-vis spectral change of **2** (1 μM in CH_2_Cl_2_, 20 °C) with (**a**) d-Lys-OMe and (**b**) l-Lys-OMe.

**Figure 7:**
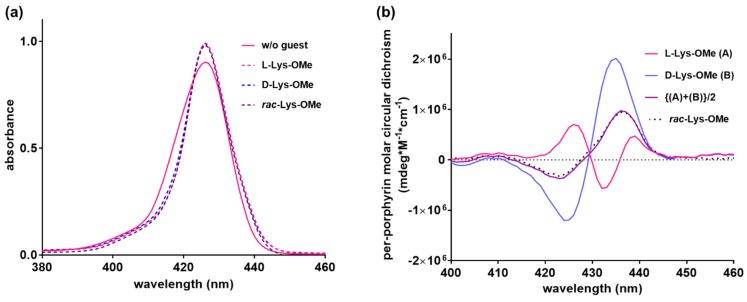
(**a**) UV-vis and (**b**) CD spectra of **2** (1 μM in CH_2_Cl_2_) with 17 equivalences of l-Lys-OMe, d-Lys-OMe or racemic Lys-OMe.

The chiral host can be utilized in the analysis of racemic guest by forming diastereomeric complex, and used practically as a NMR shift reagent to analyze racemic compounds [25]. Hence, we investigated chiral porphyrin tweezer **2** to analyze the racemic compounds based on CD and UV-vis spectroscopy. dl-, l-, and d-Lys-OMe were used as the model racemic or enantiomeric guests. First, with each pure enantiomer, the complexation of **2** was evaluated. With d-Lys-OMe, a positive Cotton effect was observed (Figure 6a); meanwhile, the ‘’M-shape” ECCD signal appeared upon binding with l-Lys-OMe (Figure 6b). As shown in the complexation of the achiral host and chiral guest complexation section, l-Lys-OMe induced a negative Cotton effect with **1** (Figure 2). Chiral **2** showed the positive Cotton effect without any guest (Figure S1). Owing to the opposite preference of peptoid backbone and guest amino acid for the orientation of co-facial porphyrins, a diminished ECCD signal is observed (Figure 6b). In addition, the M-shape ECCD signal of **2** with l-Lys-OMe (equivalence higher than 1) suggests the possible equilibrium of two (or more) conformers giving different exciton coupling signals [24]. With the highest equivalents of l- and d-Lys-OMe, **2** did not exhibit the mirror image of ECCD (Figure 7b), unlike achiral **1** that exhibited the mirror image shown in Figure 2b. These non-mirror images of the ECCD spectra indicate that the host–guest complexes (i.e., **2** with l-Lys-OMe and **2** with d-Lys-OMe), are diastereomeric to each other.

UV-vis and CD spectra were measured using racemic amino acid complexed with **2**. As shown in Figure 7a, l-, d-, and racemic samples provided the same UV-vis spectra; however, ECCD demonstrated all distinct spectral signatures. Interestingly, the value of [(A) + (B)]/2 was almost the same as the measured CD value of **2** complexed with racemic Lys-OMe (Figure 7b).

## 3. Materials and Methods

### 3.1. Abbreviations

Trifluoroacetic acid (TFA); triisopropylsilane (TIS); *N*,*N-*dimethylformamide (DMF); *N*,*N′*-diisopropylcarbodiimide (DIC); *N*-methyl-2-pyrrolidone (NMP); acetonitrile (ACN); 5-(4-carboxyphenyl)-10,15,20-triphenylporphyrin *N*-hydroxysuccinimide ester (TPP-NHS); methanol (MeOH); benzylamine (*N*pm); (*S*)-(−)-1-phenylethylamine (*N*spe). 

### 3.2. Materials and Reagents

The solvents and reagents were purchased from Sigma-Aldrich (St. Louis, MO, USA), Acros Organics (Geel, Belgium), Alfa Aesar (Haverhill, MA, USA), or TCI (Tokyo, Japan) and used without further purification. Rink amide MBHA resin (typical loading level 0.52 mmol/g), DMF and NMP were purchased from Merck Millipore (Darmstadt, Germany). Bromoacetic acid, TIS, piperidine, CH_2_Cl_2_, benzylamine, (*S*)-(−)-1-phenylethylamine, Zn(OAc)_2_, Cu(OAc)_2,_ pyridine, 4,4′-dipyridyl, 4,4′-trimethylene-dipyridine, ethylenediamine, 1,4-diaminobutane, 1,6-diaminohexane, piperidine, 4,4′-bipiperidine, 4,4′-trimethylenedipiperidine, cysteamine, 1,4-butanediol and Amberlyst A21 free base were purchased from Sigma Aldrich. DIC, l-Lys-OMe∙2HCl, d-Lys-OMe∙2HCl, and *i*-Pr_2_NEt were purchased from TCI. Acetonitrile (ACN) and TFA were purchased from Acros Organics. Oasis^®^ 6cc Vac cartridge HLB (60 μm (LP), 6cc/500 mg) was purchased from Waters (Milford, MA, USA).

### 3.3. Synthesis of MPPCs

Porphyrin-peptoid conjugates were synthesized by following a previously reported method (Scheme 1) [14,15]. Zinc and copper ions were metallated on the porphyrin peptoid conjugates. Zinc incorporation was performed using zinc acetate (Zn(OAc)_2_, 40 equiv., 64 μmol, 12 mg), *i-*Pr_2_NEt (129.25 g/mol, 7 equiv., 11 μmol, 14 mg) and peptoid (1.6 μmol) in 3 mL of CH_2_Cl_2_/MeOH (3:1, *v*/*v*). The reaction mixture was stirred continuously overnight, and then, excess zinc acetate was removed by water extraction. Copper ion incorporation was performed using copper acetate (Cu(OAc)_2_, 20 equiv., 65 μmol, 12 mg) and peptoid (3.3 μmol after passing a pipette containing silica to remove TFA salt of peptoid) in CH_2_Cl_2_. The stock solution of 0.1 M Cu(OAc)_2_ was prepared in MeOH. The reaction mixture was stirred continuously overnight at room temperature. The crude metallated porphyrin peptoid conjugates were purified using solid-phase extraction (SPE). Hydrophilic lipophilic balance (HLB) SPE cartridge (Sorbent: Oasis^®^ HLB 60 μm (LP), 6cc/500 mg) was used. After equilibration with 3 mL water, the sample dissolved in 10% MeOH (in water) was loaded. A wash was followed by 10 mL methanol to remove excess salts. The 4 mL MeOH/CH_2_Cl_2_ 1:3 *v*/*v* and 5 mL CH_2_Cl_2_ was eluted, and the fractions were collected and analyzed using LC-MS.

### 3.4. Liquid Chromatography-Mass Spectrometry (LC-MS)

The purity of the product was confirmed by using an LC-MS system. The LC and mass data of peptoids were obtained from an Agilent LC-MS system (Agilent Technologies, Santa Clara, CA, USA) that is equipped with 1260 Infinity LC (Agilent Technologies, Santa Clara, CA, USA) and 6120 SQ bundle system (Agilent Technologies, Santa Clara, CA, USA) with an atmospheric pressure ionization (API)-electrospray ion source (Agilent Technologies, Santa Clara, CA, USA). A Poroshell 120 EC-C18 (2.7 μm, 4.6 mm × 50 mm) column (Agilent Technologies, Santa Clara, CA, USA) was installed with the LC-MS system. Because each synthesized compound exhibited different solubilities depending on the LC solvents, each compound was analyzed under different conditions (detailed conditions are described in Figure S1). The mass data were obtained with the positive mode (Table S1).

### 3.5. Desalting of d/l-Lys-OMe∙2HCl

To employ d/l-Lys-OMe as a model chiral guest, desalting of d/l-Lys-OMe∙2HCl was performed using Amberlyst A21. Further, 600 mg of Amberlyst A21 was washed with MeOH and water; subsequently, 100 mg of d/l-Lys-OMe∙2HCl dissolved in 3 mL ethanol/water (5:1, *v*/*v*) was added into Amberlyst A21. After 1 h, the solution was filtered and evaporated. The obtained free d/l-Lys-OMe was used in spectroscopic titration.

### 3.6. Spectroscopic Titration

CD spectra and UV-vis spectra were obtained by adding the guest to a sample containing MPPCs continuously. CD and UV-vis spectra were recorded using a Jasco model 810 spectrometer (Jasco, Inc., Easton, MD, USA) with the scan speed of 100 nm/min, 1 nm data pitch, and band width of 1 nm. All spectra were averaged over three measurements with a 1 cm path length cuvette. As a representative example, CD and UV-vis spectra of 2 mL of 0.27 μM **2** in CH_2_Cl_2_ was obtained with the sequential addition of 1 mM or 0.1 mM guest solution. The guests were dissolved in 0.27 μM **2** in CH_2_Cl_2_ to prohibit the dilution of the host samples. The concentration of the guest stock solution was adjusted accordingly based on the pKa of the guest.

### 3.7. Calculation of Dissociation Constant

The dissociation constant of the complexation was evaluated by applying a nonlinear curve fitting with an equation used in the previous porphyrin tweezer study into the binding isotherm from spectroscopic titration [23,69]. In this study, the spectral change of UV-vis absorbance at 416 nm was plotted along with the added guest concentration (Figure S4). Subsequently, the obtained plot was fitted with the nonlinear curve equation of 1:1 binding mode (Equation (1)), where [G] and [H] represent the total concentration of the host and guest, respectively. The parameter $A_{sat}$ implies the absorbance at 416 nm when the complexation is saturated:

(1) $$\Delta A_{416\;nm}=\left(A_{sat}\left(K_{d}+\left[G\right]+\left[H\right]\right)\;\;\;\;\;-\sqrt[{2}]{(A_{sat}^{2}{\left(\left[G\right]+\left[H\right]+K_{d}\right)}^{2}-4A_{sat}^{2}\left[G\right]\left[H\right]}\right)/2\left[H\right]$$

## 4. Conclusions

In this work, we incorporated metal ions (Zn^2+^ and Cu^2+^) in co-facial porphyrins present on a peptoid backbone to construct chiral or achiral metalloporphyrin tweezers. First, the chirality transfer from the d- or l-amino acid guest to achiral porphyrin tweezer **1** was observed. Using chiral porphyrin tweezer **2**, a broad range of achiral dinitrogen guests with different lengths and basicities were complexed. The longest and most basic guest tested in this study showed the lowest dissociation constant value (K_d_). With a racemic amino acid sample, chiral **2** formed a mixture of two diastereomeric complexes (**2** + d-Lys-OMe and **2** + l-Lys-OMe). When compared to copper-incorporated MPPCs (**3**), the zinc-incorporated MPPCs (**1** and **2**) exhibited clear spectral changes that helped in the recognition of complexation. The zinc porphyrin-peptoid conjugates, as a novel class of porphyrin tweezer, pose several unique aspects, taking advantage of structural characteristics of backbone peptoids: (1) inter-porphyrin distance can be changed, (2) chirality can be modulated via peptoid helicity modulation, and (3) flexibility between two porphyrins can be tuned. The development of finely-engineered porphyrin tweezers using the MPPC platform is currently a topic of our study, which can potentially be used as sensors, catalysts or delivery carriers.

## Figures and Tables

**Table 1:** Results of spectroscopic titration for binding constant determination (complexation of **2** with guest molecules).

Guests	Length (Å)	K_d_ (10^−6^ M)	R^2^	ECCD λ_max_/λ_min_ (nm) ^a^	UV-vis λ_max_ (nm) ^a^
Without guest	-	-	-	430/423	427
Pyridine ^b^	3.8	1.5	0.84	430/423	428
4,4′-Dipyridyl	6.9	7.9	0.99	Nd ^c^	427
4,4′-Trimethylenedipyridine	10	0.83	0.98	433/427	427
Ethylenediamine	3.8	0.83	0.94	Nd ^c^	424
1,4-Diaminobutane	6.3	0.55	0.99	434/423	426
1,6-Diaminohexane	8.8	0.074	0.99	435/427	427
Piperidine	3.9	9.2	0.99	434/425	430
4,4′-Bipiperidine	7.3	0.10	0.99	Nd ^c^	427
4,4′-Trimethylenedipiperidine	9.8	0.019	0.99	435/428	429
Cysteamine	4.1	0.95	0.99	435/427	427

^a^ With the highest equivalence of guest, the spectral maximum and minimum were recorded. ^b^ No isosbestic point was observed. ^c^ The maximum and minimum of the ECCD spectra could not be determined.

## Supplementary Materials

The following are available online, Figure S1: LC-MS chromatograms of compound **1**–**3** with UV detection at 220 nm, Figure S2: Temperature dependent CD spectra (**a**) and UV-vis spectra (**b**) of **2** (0.27 μM) in CH_2_Cl_2_, Figure S3: CD and UV-vis spectroscopic titration of **2** with dinitrogen guests, Figure S4: A representative non-linear fitting for the host-guest titration of **2** with 4,4′-dipyridyl, Figure S5: The CD and UV-vis spectral change of **3** (0.27 μM in CH_2_Cl_2_) with 4,4′-dipyridyl and 1,4-butanediol, Table S1: MS data of metalloporphyrin-peptoid conjugates.
