# Supplementary material for: Metalloporphyrin Dimers Bridged by a Peptoid Helix: Host-Guest Interaction and Chiral Recognition
Source: Molecules. 2018 Oct 24;23(11):2741. doi: 10.3390/molecules23112741 (PMC6278558; doi:10.3390/molecules23112741)
Supplement: Supplementary file 1 [file molecules-23-02741-s001.pdf]

*Supplementary Material*

# Metalloporphyrin Dimers Bridged by a Peptoid Helix: Host-Guest Interaction and Chiral Recognition

**Yen Jea Lee, Boyeong Kang and Jiwon Seo ***

Department of Chemistry, School of Physics and Chemistry, Gwangju Institute of Science and Technology, Gwangju 61005, Korea; leeyeonjae@gist.ac.kr (Y.J.L.); boyeongkang2022@u.northwestern.edu (B.K.)

* Correspondence: jseo@gist.ac.kr; Tel.:+82-62-715-3675; Fax /+82-62-715-2866

## Contents

**Figure S1.** LC-MS chromatograms of compound **1–3** with UV detection at 220 nm.
**Figure S2.** Temperature dependent CD spectra (**a**) and UV-vis spectra (**b**) of **2** (0.27 μM) in $CH_2Cl_2$.
**Figure S3**. CD and UV-vis spectroscopic titration of **2** with dinitrogen guests.
**Figure S4.** A representative non-linear fitting for the host-guest titration of **2** with 4,4′-dipyridyl.
**Figure S5.** The CD and UV-vis spectral change of **3** (0.27 μM in $CH_2Cl_2$) with 4,4′-dipyridyl and 1,4-butanediol.

**Table S1.** MS data of metalloporphyrin-peptoid conjugates.

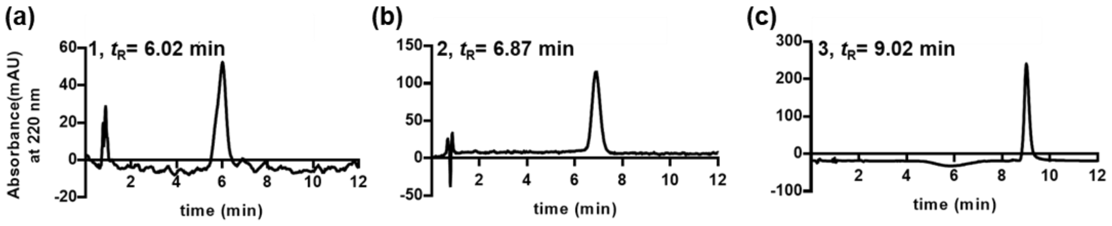


**Figure S1.** LC-MS chromatograms of compound **1**–**3** with UV detection at 220 nm. (**a**) And (**b**) isocratic elution of MeOH (0.1% TFA) was used at 30 °C. (**c**) Two solvents of isopropyl alcohol (A, 0.1% TFA) and water (B, 0.1% TFA) were used, and the concentration of A was raised from 50% to 100% for 3 minutes and maintained for 15 minutes at 30 °C.

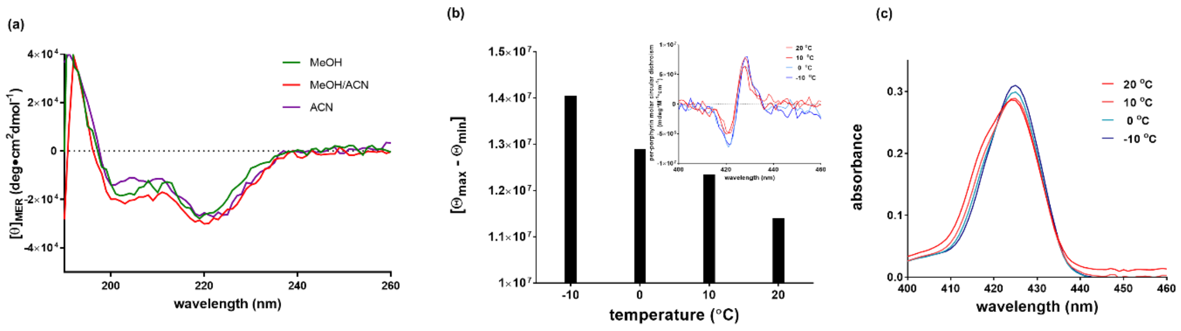


**Figure S2.** (**a**) CD spectra of 2 (50 μM) of 190–260 nm, in MeOH, ACN or MeOH/ACN=1/1 (*v*/*v*), (**b**) Temperature dependent ECCD difference, inlet: CD spectra of 2 (0.27 μM) in $CH_2Cl_2$, (**c**) Temperature dependent UV-vis spectra of 2 (0.27 μM) in $CH_2Cl_2$.

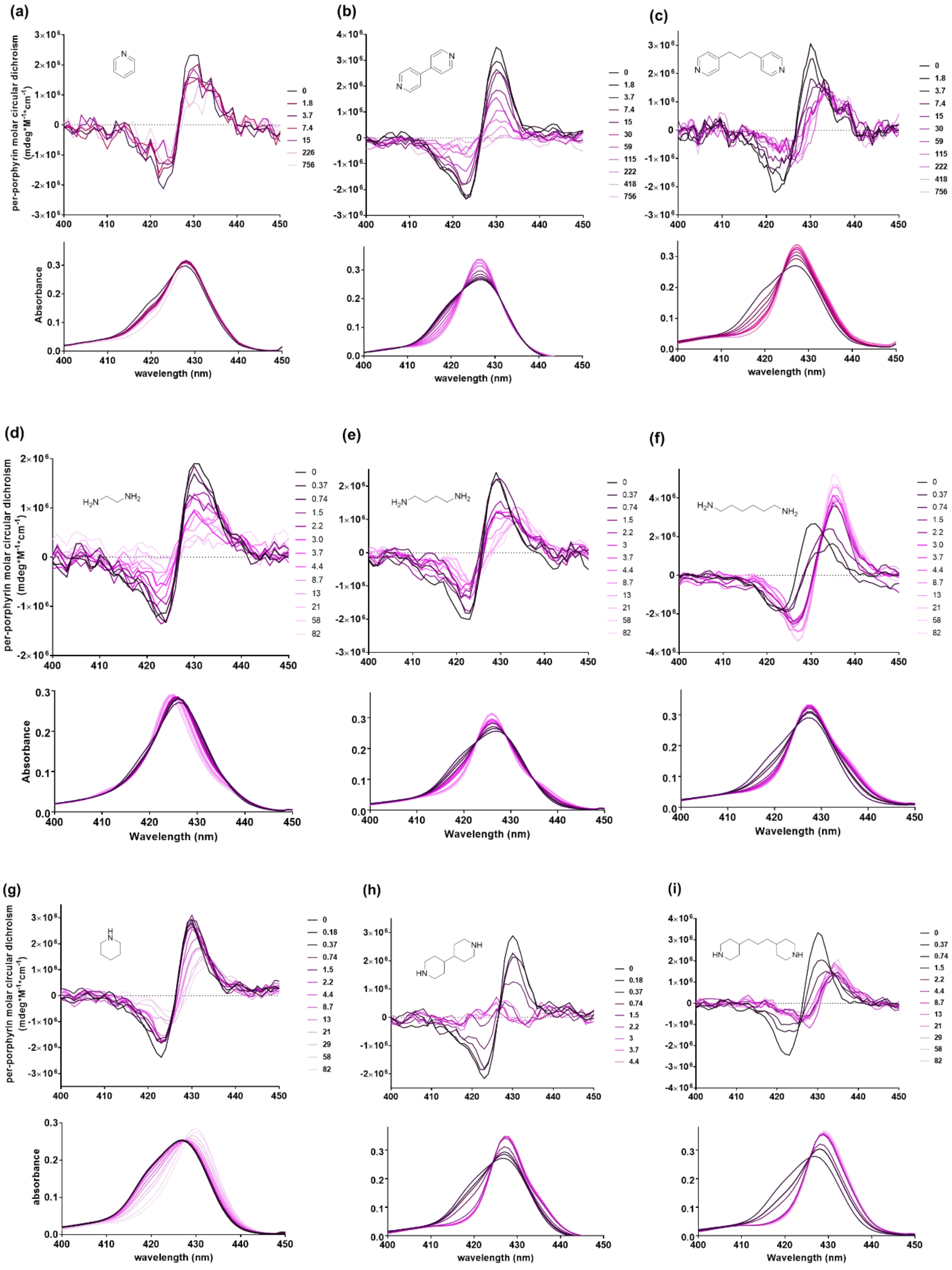


**Figure S3**. CD and UV-vis spectroscopic of **2** with dinitrogen guests. Legend on right side of each graph means the added equivalents of guest toward host.

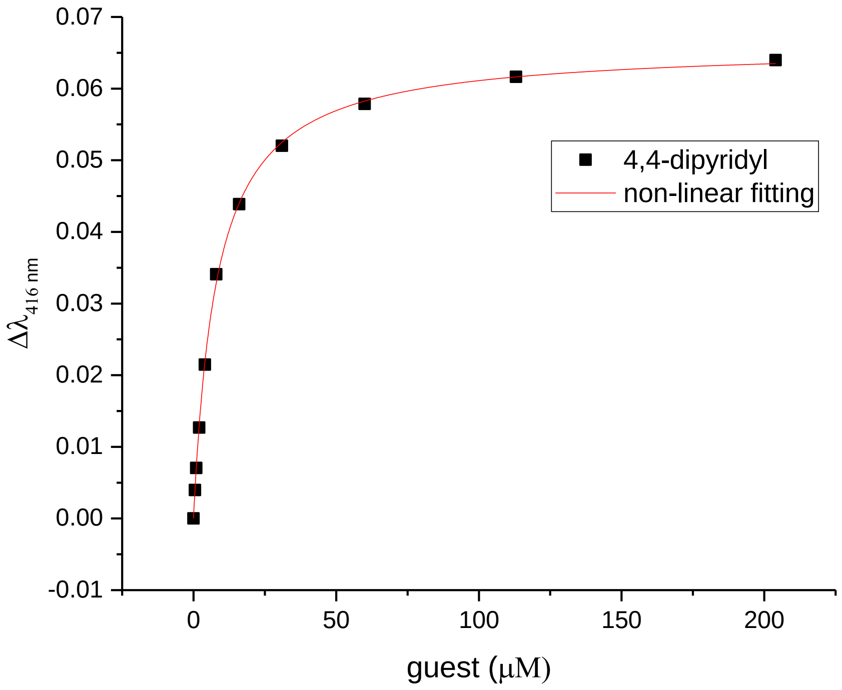


**Figure S4.** A representative non-linear fitting for the host-guest titration of **2** with 4,4′-dipyridyl.

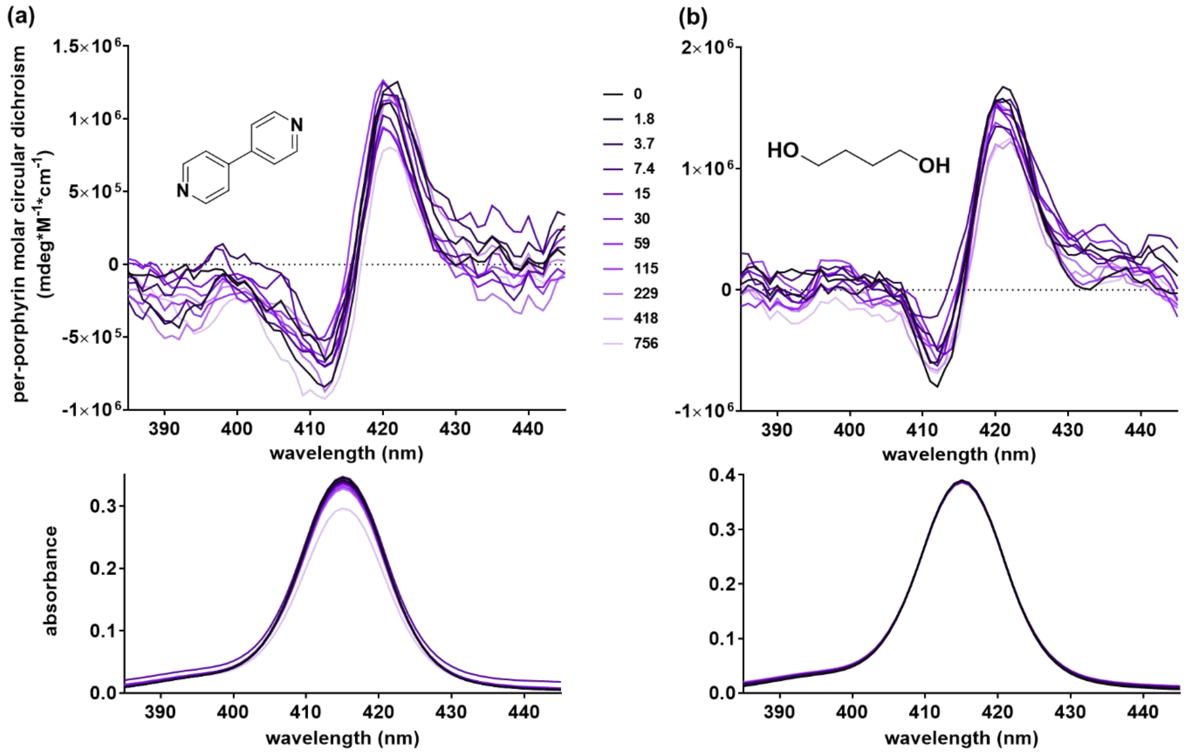


**Figure S5.** The CD and UV-vis spectral change of **3** (0.27 μM in $CH_2Cl_2$) with 4,4′-dipyridyl and 1,4-butanediol. Legend in graphs indicates the equivalents of added guest toward **3**.

**Table S1.** MS data of metalloporphyrin-peptoid conjugates

| Compound | Mass Calculated | Mass Observed (*m*/*z*) [a] |
|---|---|---|
| **1** | 2748.99 | 1375.0 $[M+2H]^{2+}$ |
| **2** | 2847.10 | 1424.7 $[M+2H]^{2+}$ |
| **3** | 2845.10 | 1422.6 $[M+2H]^{2+}$ |

[a] due to the mass detection limitation of 2000 (*m*/*z*), doubly charged species were observed.
